# Baduanjin exercise for balance function in community-dwelling older adults with cognitive frailty: a randomized controlled trial protocol

**DOI:** 10.1186/s12906-022-03764-1

**Published:** 2022-11-17

**Authors:** Yu Zhang, Jiawei Wu, Xiaoqian Wang, Guohua Zheng

**Affiliations:** 1 Shanghai University of Medicine and Health Sciences, Shanghai University of Traditional Chinese Medicine, Shanghai, 201203 China; 2 College of Nursing and Health Management, Shanghai University of Medicine & Health Sciences, Shanghai, 201318 China

**Keywords:** Baduanjin exercise, Cognitive frailty, Randomized controlled trial, Balance function, Study protocol

## Abstract

**Background:**

Balance function provides a physiological link between the physical and cognitive function, and is a potential predictor for cognitive frailty. As a gentle mind–body exercise, Baduanjin can develop flexibility and co-ordination, thus would be is helpful for the improvement of balance function. This trial will evaluate the effect of Baduanjin on balance function in older adults with CF.

**Methods/design:**

A total of 72 community-dwelling older adults with CF will be recruited and randomly allocated (1:1) into the Baduanjin exercise group or usual physical activity control group. All participants will undergo a health education program on nutrition and diet-related knowledge for 6 sessions (30 min per session) during the intervention period. Moreover, participants in the Baduanjin exercise group will receive a 24-week Baduanjin training course of 60 min per session and 3 sessions per week, while those in the usual physical activity control group will be required to maintain their original physical activity. Primary and secondary outcomes will be measured at baseline and after the 24-week intervention period. A mixed linear model will be constructed to analyse the intervention effects.

**Discussion:**

This protocol presents an objective design of a randomized, single-blind trial that will evaluate the effectiveness and safety of traditional Chinese mind–body exercise Baduanjin training on the balance ability of community-dwelling older adults with cognitive frailty. If the results are as expected, this trial will provide evidence of the effect of Baduanjin exercise on balance in an older community-based population.

**Trial registration:**

This trial was registered in the Chinese Clinical Trial Registry with code ChiCTR2100050857 and was approved on 5 September 2021.

## Background

Cognitive frailty (CF) is an important syndrome characterized by the simultaneous presence of mild cognitive impairment and physical frailty in the absence of dementia [1]. It is reported that the prevalence of CF among older population ranges from 4.4% to 9.8% in China [2]. CF can lead to accelerated decline in cognitive and physical function, resulting in a reduction in ability of daily living and quality of life, is more predictive of poor health outcomes of older adults, such as depression, hospitalization, incapacity, dementia and even mortality in older people [3].

Balance relies on signals and feedback from the vestibular system and brain. It therefore provides a physiological link between the physical and cognitive function [4, 5]. Physical and cognitive decline in older adults with cognitive frailty could lead to progressive impairment of muscle strength and coordination, further disrupting balance function [6]. It has been reported that almost 30% of older adults with frailty suffer from balance impairment [7]. However, human balance is a complex multidimensional concept related to postural control, which is essentially the ability to maintain a posture, move between postures and not fall when reacting to an external disturbance [8]. Balance ability provides the foundation for mobility and functional independence for older adults and is an important predictor of cognitive decline and physical frailty in older adults [9, 10].

Exercise is believed to have multiple benefits on improving the balance function and physical functionality of older adults [11, 12]. Regular physical exercise in older adults is associated with increased functional independence, muscle strength, bone and joint health, and improvements in balance function [13]. Therefore, physical activity guidelines recommend that adults aged over 65 years should incorporate multicomponent physical activity to improve balance at least two days a week [14]. However, most older adults can not meet national physical activity or physical exercise recommendations due to the limitation of health and environmental conditions [15, 16]. Baduanjin is one of the most widely practised forms of traditional Chinese mind-body exercise. It consists of eight separate movements with mild to moderate exercise intensity, and is characterized by symmetrical body postures and movements, breathing control, a meditative state of mind, and mental focus, thus developing the flexibility and co-ordination of the practitioner [17]. Baduanjin exercise emphasises mind-body integration to improve both physical and mental health, so it is recommended for older persons in China [18, 19]. Studies indicate that Baduanjin has positive physiological and psychological effects for older adults with/without chronic disease, for example, improving cognitive function for older adults with or without cognitive impairment [20], alleviating cancer-related fatigue in cancer patients [21], and alleviating musculoskeletal pain in older people with chronic low back pain [22]. A recent preliminary study assessing the feasibility and efficacy of Baduanjin for prefrail/frail community-based older adults found that Baduanjin training was safe and feasible for prefrail/frail older adults with the potential to improve physical and cognitive function, reduce fall risk, improve psychological well-being, and reverse frailty status [23]. Two other studies also showed that 8-12 weeks of Baduanjin training was effective in improving balance, leg strength and mobility and was a safe and sustainable form of home-based exercise for people with chronic stroke or community-dwelling older adults [24, 25]. However, the effect of Baduanjin on balance in community-dwelling older adults with cognitive frailty requires additional investigation. The main purpose of this trial is to evaluate the effect of a 24-week Baduanjin exercise intervention on balance in community-dwelling older adults with cognitive frailty. We will investigate whether a 24-week Baduanjin exercise training protocol improves static, dynamic and overall balance ability compared with a nonspecific exercise intervention group as well as investigate the safety and feasibility of this intervention protocol. The secondary objectives are to investigate the effect of this intervention protocol on cognitive ability, physical frailty, risk of falling and muscle strength.

## Methods

This study is designed and will be conducted and reported according to the Consolidation Standards of Reporting (CONSORT) 2010 Statement regarding randomized controlled trials [26].

### Study design

**Fig. 1:**
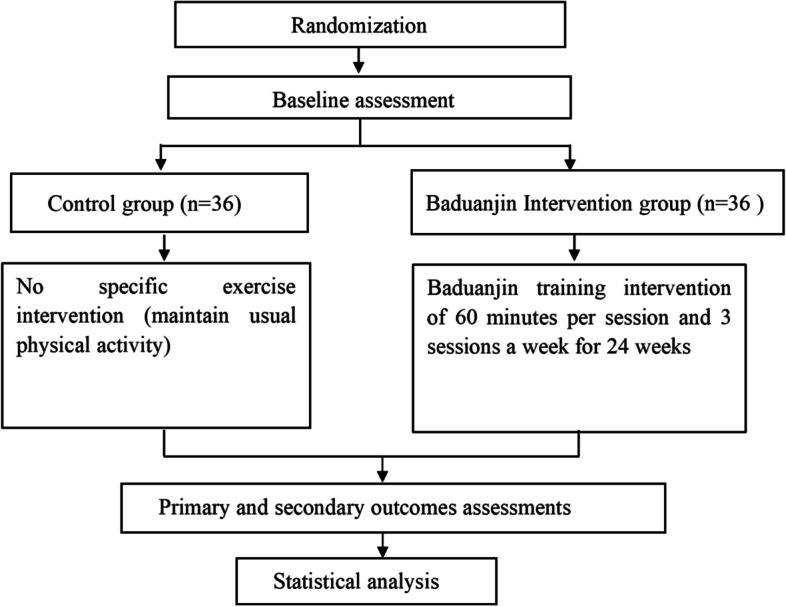
Flow chart of the trial

**Fig. 2:**
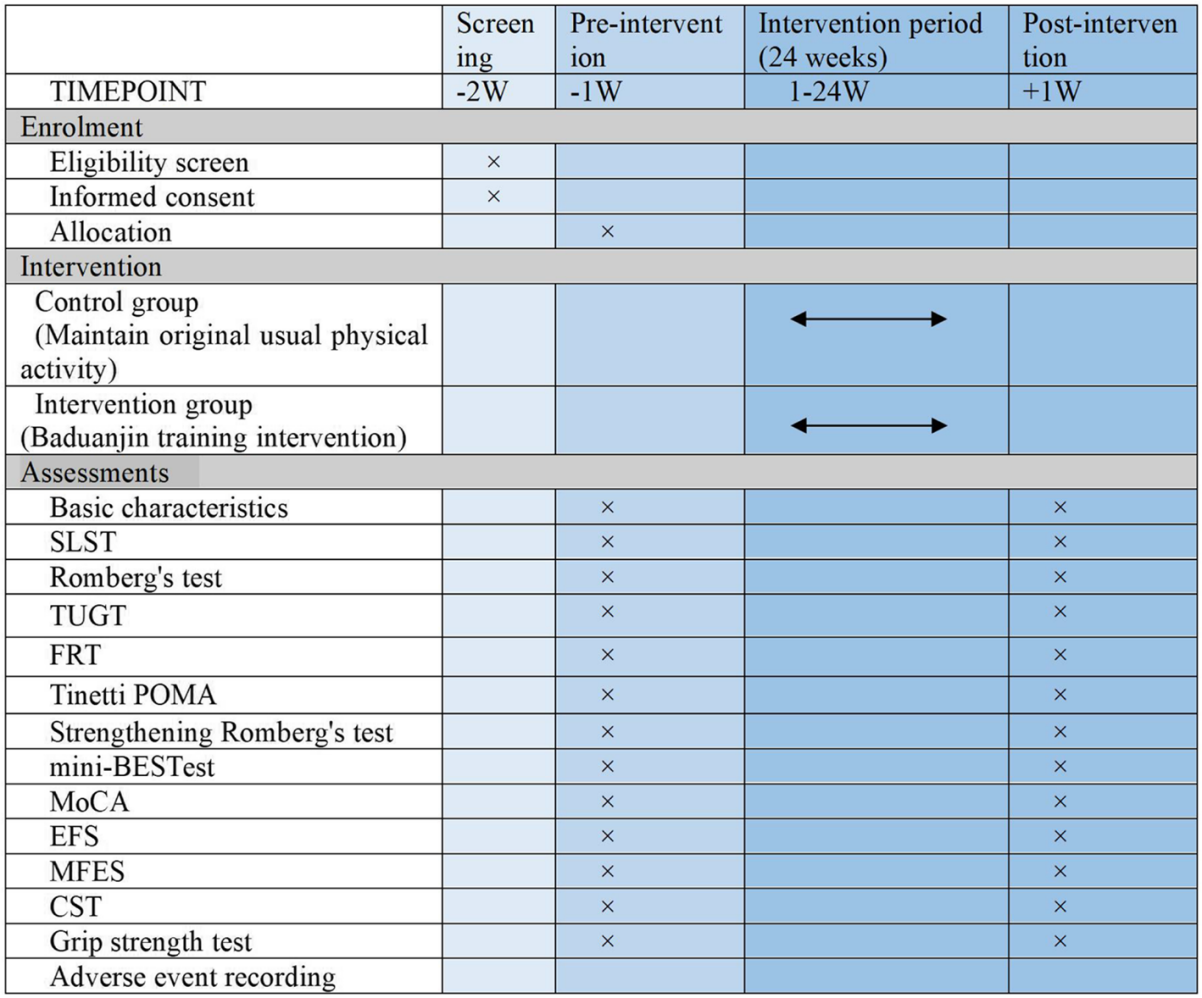
Schedule of enrolment, assessments and interventions. The × indicates at which point of the trial the respective assessments will take place. SLST: Single-leg stance test; TUGT: Timed Up and Go test; FRT: Functional reach test; Tinetti POMA: Tinetti Gait and Balance Scale; mini-BES Test: Mini Balance Evaluation System Test; MoCA: Montreal Cognitive Assessment Scale; EFS: Edmonton Frailty Scale; MFES: Modified Fall Efficacy Scale; CST: the 30-s chair-stand test

This is a prospective, randomized controlled trial with blinded outcome measures comparing traditional Chinese mind–body exercise Baduanjin intervention versus nonspecific exercise intervention (maintaining usual physical activity) for community-dwelling older adults with cognitive frailty (CF). A total of 72 older adults with CF will be randomly (1:1) allocated into the Baduanjin intervention group (60 min per session, three sessions per week for 24 weeks) and the control group (maintaining their original usual physical activity). The study procedure and outcome assessment schedule for this trial are presented in Figs. 1 and 2. This trial was registered prospectively at Chictr.org.cn with registration number ChiCTR2100050857 on 5 September 2021.

## Sample size calculation

Sample size was calculated based on the group mean comparison formula by using the effect size of the primary outcome (mini-BESTest scores) from the study conducted by Ni M et al. [27]. Considering 80% efficacy and 5% I type error (two tails), at least 30 subjects in each group were enough to detect the target effect mini-BESTest changes between two groups. Considering a 20% dropout rate, 36 subjects each group are planned to be recruited in this trial. Calculations were performed using Gpower 0.3.1.6.2 software.

## Study population

### Eligibility criteria

Inclusion criteria: (1) community-dwelling older adults aged over 60 years; (2) meet the CF criteria [28]; (3) no regular physical exercise within half a year (regular exercise defined as a frequency of at least 20 min at least twice a week); (4) written informed consent.

Exclusion criteria**:** (1) those suffering from uncontrollable blood pressure (systolic blood pressure ≥ 160 mmHg or diastolic blood pressure ≥ 100 mmHg); (2) with alcohol or drug abuse; (3) with exercise contraindications such as mental illness, depression, coronary artery disease, musculoskeletal disorders, etc.; (4) joining other trial.

### Recruitment

Eligible older adults will be recruited from three community centres (Xingshengli, Huashengli, Zhongjinhaitangwan) in Pudong New District in Shanghai city in China. The potential participants can obtain specific recruitment information through a combination of online and offline way, including the community health service centre, community health management system, community health lectures, WeChat and the internet. They will be first assessed for eligibility by two research assistants according to the inclusion and exclusion criteria, and then the informed consent will be discussed if they are interested in this trial. After voluntarily signing the informed consent form, he or she will be invited to participate in the trial. Recruitment starts on 1 March 2021 and end when 72 participants are enrolled.

### Baseline assessment

Baseline information of all eligible participants will be collected by the recruiters using a self-designed questionnaire. Baseline information includes the participants’ personal general data and demographic information, such as gender, age, height, weight, years of education, marital status, occupation, medical history, lifestyle, diet, and physical activity.

### Randomization, allocation concealment, and blinding

The programs of randomization and allocation concealment will be performed as reported in our previous trail protocol [29]. Although it is difficult to blind the participants in this trial, the outcome assessors and statisticians will be blinded according to the program described in our previous trial protocol [29].

## Intervention protocol

All participants will participate in a health education program on nutrition and diet-related knowledge of cognitive frailty, which will consist of lectures (30 min per session once every 4 weeks) and knowledge booklets (every 4 weeks) during the intervention period.

### Control group

Participants in the control group will not receive any specific exercise training (i.e., maintain their original usual physical activity).

### Baduanjin exercise training group

Participants in the Baduanjin exercise training group will receive Baduanjin exercise training with a frequency of 3 days a week and 60 min a day for 24 weeks. Baduanjin practice will be conducted at three community centres (Xingshengli, Huashengli and Zhongjinhaitangwan) with 10–15 individuals per centre. Three professional teachers who have been occupied in the Baduanjin teaching at the Shanghai University of Medicine & Health Sciences (SUMHS) for at least 5 years will be employed to teach participants’ Baduanjin practice. The training scheme of Baduanjin exercise is planned as described in detail previously [29].

## Outcome measures

In this trial, the primary and secondary outcomes will be measured at baseline (1–2 weeks before randomization) and at the end of the 24-week intervention period (25 weeks after randomization). All outcome assessments will be independently conducted by experienced and blinded assessors. A summary of all measurements in this trial is shown in Fig. 2.

### Primary outcome measure

The primary outcome is balance ability measured using the Tinetti Performance-Oriented Mobility Assessment (Tinetti POMA) and the Mini Balance Evaluation System Test (Mini-BESTest).

The Tinetti POMA scale contains both a balance and a gait component. The balance component of the test assesses the individual’s ability to maintain postural control, and the gait component assesses the symmetry, continuity, path, base of support, and postural sway during gait [30]. The Tinetti POMA has been widely used to screen balance and gait abnormalities in the community or clinical population, with well-demonstrated reliability and validity [31]. The Mini-BESTest includes four sections, anticipatory postural adjustments (APA), reactive postural control (RPC), sensory orientation (SO) and dynamic gait (DG), and consists of 14 items that examine performance tasks related to dynamic balance, obstacle negotiation, reactions to external forces, and performance during dual tasking with cognitive challenge [32].

### Secondary outcome measures

- Static balance ability will be measured by the single-leg stance test (SLST) and Romberg’s test.
- Dynamic balance ability will be measured by the Timed Up and Go test (TUG) and functional reach test (FRT).
- Cognitive ability will be measured by the Chinese version of the Montreal Cognitive Assessment scale.
- Physical frailty will be measured by the Chinese version of the Edmonton Frailty Scale.
- Fear of falling will be measured by the Modified Fall Efficacy Scale.
- Lower extremity muscle strength will be measured by the 30-s chair stand test (CST).
- Hand grip strength will be measured by an electronic grip force meter.

The SLST is widely used to assess static balance ability in older adults and has normative data accepted by the scientific literature [33]. Romberg’s test evaluates balance ability by having individuals stand as much as possible without deviating from the standing position, regardless of foot position and different visual conditions (i.e., eyes open/eyes closed) [34].

The TUG test is utilized for the evaluation of functional mobility involving velocity, potency, and dynamic balance and is a common test to assess gait stability and balance in older adults [35], while the FRT is also a valid test in the assessment of balance in older adults by measuring the limits of stability of individuals while reaching forward in a standing position, in which the limits of stability are defined as the maximum distance that the centre of mass can be moved safely without changes in the base of support [36].

The Chinese version of the Montreal Cognitive Assessment Scale (MoCA) is a brief cognitive screening test that covers eight cognitive domains, including visual spatial ability and execution, naming, memory, attention, language, abstraction, delayed recall, and orientation, with good validity and sensitivity in the Chinese population [37].

The Edmonton Frailty Scale (EFS) consists of nine domains, such as cognition, basic health, independence, social support, drug use, nutrition, mood, control, and function performance, with good interrater reliability (k = 0.77) [38].

The Modified Falls Efficacy Scale (MFES) will be used to assess fear of falling by measuring confidence in performing a range of specific activities of daily living associated with an increased risk of falling. It is a well-established measure with acceptable reliability, validity, and sensitivity in different older people [39].

The CST is a functional test of lower extremity muscle strength of older adults that measures their ability to rise from a chair and is also involved in other functional domains, such as balance, coordination, and endurance [40].

Hand grip strength is an indicator of upper body strength and is also an indicator of overall muscle strength in the community-dwelling older population [41], which will be measured using an electronic grip force meter (CAMRY-EH101).

## Adverse events

Participants will be supervised for adverse events during the intervention. The research assistant will describe and report the adverse events to research group by the adverse event report form. The medical monitor and research group will evaluate each adverse event and classify it by severity (mild, moderate, severe/undesirable, potentially life threatening or death) and by grading (unrelated, possibly related, or definitely related). If serious adverse events occur, it will be reported to the ethics committee immediately.

## Statistical analysis

Data will be analysed according to the intention-to-treat principle (ITT) using the SPSS 21.0 software package by an independent statistician, with a statistical significance of *p* < 0.05 on both sides. Missing data will be complemented using the multiple imputation method.

Descriptive statistics will be performed using the mean and standard deviation (SD)/median and interquartile ranges for continuous outcomes or percentages for categorical outcomes. Baseline characteristics will be compared between the Baduanjin exercise training group and the control group to identify the adequacy of randomization and possible confounders. We plan to analyze primary and secondary outcomes using the mixed-effect linear model with restricted maximum likelihood, and group (Baduanjin exercise training and the control group), time (baseline and after intervention) and possible confounding factors will be introduced into the model as independent variables.

## Data collection and management

Data will be recorded by the outcome assessors using the case report from (CRF) and then transcribed into the electronic data acquisition system (EDC) by the research assistants. This EDC system is a free research manager platform (ResMan, http://www.medresman.org) complied with current safety standards, which is managed by the China Clinical Trial Registry. The data will be saved in the EDC system with a separate password-protected location.

## Dissemination

The study protocol is in accordance with the Helsinki Declaration and can be obtained through the Chinese Registry website (registered on ChiCTR.org with the identifier ChiCTR2100050857). The results of the study will be published in peer-reviewed science journals or presented at local, national, or international conferences. The findings of this study will also be shared directly with all participants and disseminated to researchers, health service providers, health care professionals and the general public through courses, presentations, and the internet.

## Discussion

Cognitive decline and physical frailty are two major clinical and public health challenges among community-dwelling older adults. An increasing number of studies have shown that either physical frailty or cognitive impairment is significantly associated with a higher risk of falls, suggesting that fall prevention might be beneficial for cognitive frailty prevention [42–44]. As one of the most popular traditional Chinese exercises, Baduanjin exercise has been practised to promote health in community populations in China for 1000 years [45]. Increasing evidence has also shown that Baduanjin exercises have a positive effect on improving cognitive and physical function in older adults with different health conditions [20]. Moreover, Baduanjin exercise is also a mind–body exercise of mild to moderate intensity and is easier to learn and practice with fewer limitations and less physical and cognitive demands because it only consists of eight separate and smooth movements [17]. Therefore, it is more suitable to practice for older adults. Furthermore, Baduanjin exercise places a special emphasis on a combination of symmetrical physical postures and meditative mind and breathing techniques in a harmonious manner [17]. The body needs to maintain a steady gravity centre and then drive the movement of the four limbs with the lumbar spine as the axis when practising Baduanjin exercise [46], which is potentially effective in improving gait parameters, mobility, and even balance. Therefore, we designed a randomized controlled trial (RCT) to identify the effect of regular Baduanjin training on balance ability, cognition, and physical function among community-dwelling older adults with cognitive frailty. This trial will be conducted with a rigorous method to control bias, including randomization, parallel control and blinding of the outcome assessors and statisticians. The accessible Baduanjin exercise training protocol (60 min per day 3 days a week for 24 weeks) and the extensive outcome assessment on balance function will be applied in this trial. In addition, we will also employ qualified physical exercise teachers to guide the Baduanjin training of participants. Thus, the results of this trial should be generalizable to community-based older adults with CF.

Some potential limitations are inevitable in this protocol. First, it is difficult to monitor participants' other physical activities during the intervention period. Although all participants will be required to use the pedometer in their mobile phones to record their daily physical activity or exercise information, this is still not enough to measure the intensity of their daily activities. Second, due to unexpected events, such as training time conflicting with other commitments and bad weather, these uncontrollable causes will influence the adherence to the Baduanjin training program in the Baduanjin exercise group and may affect the results of the study. In addition, because of the characteristics of the exercise intervention, it is impossible to blind the participants in this trial. Despite facing the above challenges, we will strive to conduct this trial according to its protocol.

In summary, this trial will provide health care professionals and older adults in the community, especially those with cognitive frailty, with directly applicable evidence about the effects of the traditional Chinese mind–body exercise Baduanjin for improving balance, cognitive ability, and physical function.

### Trial status

At the time of submission, recruitment for the trial has been completed. The first participant was included on 30 December 2021.

## Data Availability

Data sharing is not applicable to this article as no datasets were generated or analysed during the current study.

## Abbreviations

CF: Cognitive frailty
CONSORT: Consolidation Standards of Reporting Statement
Tinetti POMA: Tinetti Performance-Oriented Mobility Assessment
Mini-BESTest: Mini Balance Evaluation System Test
APA: Anticipatory Postural Adjustments
RPC: Reactive Postural Control
SO: Sensory Orientation
DG: Dynamic Gait
SLST: Single-leg Stance Test
TUG: Timed Up and Go Test
FRT: Functional Reach Test
CST: Chair Stand Test
MoCA: Montreal Cognitive Assessment Scale
EFS: Edmonton Frailty Scale
MFES: Modified Falls Efficacy Scale
RCT: Randomized Controlled Trial
